# Effect of Polydopamine and Curcumin on Physicochemical and Mechanical Properties of Polymeric Blends

**DOI:** 10.3390/ma16175758

**Published:** 2023-08-23

**Authors:** Muhammad Tahir, Alina Sionkowska

**Affiliations:** Department of Biomaterials and Cosmetic Chemistry, Faculty of Chemistry, Nicolaus Copernicus University in Torun, Gagarina 7, 87-100 Torun, Poland; 503438@doktorant.umk.pl

**Keywords:** polyvinyl alcohol, sodium alginate, curcumin, polydopamine, polymeric composite

## Abstract

In this study, we prepared composites made from polyvinyl alcohol (PVA), sodium alginate (SA), curcumin (Cur), and polydopamine (PD). The film-forming properties of the composites were researched for potential wound-healing applications. The structures of the polymer blends and composites were studied by FTIR spectroscopy and microscopic observations (AFM and SEM). The mechanical properties were measured using a Zwick Roell testing machine. It was observed that the formation of a polymeric film based on the blend of polyvinyl alcohol and sodium alginate led to the generation of pores. The presence of curcumin in the composite resulted in the alteration of the blend properties. After solvent evaporation, the polymeric blend of PVA, SA, and curcumin formed a stable polymeric film, but the film showed poor mechanical properties. The addition of polydopamine led to an improvement in the mechanical strength of the film and an increase in its surface roughness. A polymeric film of sodium alginate presented the highest surface roughness value among all the studied specimens (66.6 nm), whereas polyvinyl alcohol showed the lowest value (1.60 nm). The roughness of the composites made of PVA/SA/Cur and PVA/SA/Cur/PD showed a value of about 25 nm. Sodium alginate showed the highest values of Young’s modulus (4.10 GPa), stress (32.73 N), and tensile strength (98.48 MPa). The addition of PD to PVA/SA/Cur led to an improvement in the mechanical properties. Improved mechanical properties and appropriate surface roughness may suggest that prepared blends can be used for the preparation of wound-healing materials.

## 1. Introduction

Polymeric blends are widely researched by scientists for many purposes. Several blends have also been used to fabricate biomedical devices [1,2,3]. Polymeric blends for biomedical applications can be prepared based on synthetic and natural polymers. It is believed that blending two or even more polymers can lead to the development of a new class of materials that show improved mechanical properties and biocompatibility when compared with materials made of a single polymer. In this research, for the preparation of a polymeric blend, we propose polydopamine (PD), polyvinyl alcohol (PVA), and sodium alginate (SA).

Polydopamine is a synthetic biomimetic polymer, and its preparation is solely based on oxidation and polymerization of dopamine monomers. Oxidation and polymerization of dopamine monomers lead to polydopamine under alkaline conditions [4,5]. Polydopamine holds anti-oxidative, anti-tumor, and anti-bacterial properties. These properties make polydopamine highly applicable in cancer detection, drug delivery, and treating cancer tumors [6,7]. Polydopamine is widely used in biomedical applications as an adhesive layer and protective coating, as it presents low toxicity and bioconjugation [8,9]. Zhou et al. mentioned that polydopamine hydrogels have applications in preventing and treating oral diseases like oral ulcers, dentin caries, enamel caries, and peri-implantitis [10]. Polydopamine adjusts to the surface of substrates [11], presenting excellent biocompatibility and an adhesive nature [12].

Polyvinyl alcohol is a synthetic polymer that exists in a semi-crystalline form. Polyvinyl alcohol has been found to be non-toxic in the case of in-vitro and in-vivo experimental studies [13]. Millon et al. mentioned that polyvinyl alcohol has a hydrophilic nature, and it also shows biocompatibility. Polyvinyl alcohol can be easily transformed into a stable hydrogel, leading to good mechanical strength [14]. Georgieva et al. reported that polyvinyl alcohol is highly sensitive as it makes contact with moisture, so polyvinyl alcohol is highly applicable in the biomedical field [15]. Gao et al. stated that the hydrogel of polyvinyl alcohol has applications in wound healing and cell therapy [16]. Jiang et al. noted that polyvinyl alcohol holds similar deformation properties to prostate tissues and is used as a substitute for soft tissues [17] Moreover, PVA has been applied in biomedical devices as it shows low protein adsorption and presents chemical resistance. It can be also applied in cartilage replacement, as it fulfills safety standards requirements [18].

Sodium alginate (SA) belongs to the polysaccharide group of natural polymers [19]. SA is a linear polymer with good film-forming properties [20,21] and has a hydrophilic nature [22]. It is well known due to its excellent cross-linking ability, and it is also biocompatible. Sodium alginate is highly applicable in tissue repair or regeneration due to its excellent printability [23]. Shaikh et al. claimed that SA has proven applications in cancer treatment as it is used for cancer drug delivery vaccines and has a biodegradable nature [24]. Sodium alginate has been used in wound-healing applications as it has a similar extracellular structure to living organisms, and its maintenance at lower temperatures is easy [25,26]. Zdiri et al. mentioned blending alginate with other materials to achieve wound-dressing applications [27].

The blends of SA and PVA have been already considered for biomedical applications. Bahadoran et al. utilized sodium alginate and polyvinyl alcohol, and it has been observed that polymeric blends of SA and PVA enhanced the skin wound-healing rate in rat models. Bahadoran et al. noticed that the polymeric blend effectively affected cell proliferation. A polymeric blend of PVA and SA led to a decreased wound size, which means that it increased the percentage of wound closure [28]. Cell proliferation is defined as the increase in the number of cells caused by the division of the cell [29]. Cell proliferation is found to be effective in wound healing related to gastrointestinal and dermal wounds. Even in skin repair, it has a pivotal role. Cell proliferation plays a crucial role in wound healing, as it is associated with the controlled cell system [30,31]. Tumorigenesis leads to the disorganization of the cell system, and cancer results because of uncontrolled cell proliferation [32]. Saraiva et al. noticed an improvement in wound-healing material’s mechanical, physiochemical, and biological properties when a blend of sodium alginate and polyvinyl alcohol was used instead of individual polymers in wound-healing material [33]. Muangsri et al. noted that a polymeric blend of sodium alginate and polyvinyl alcohol led to a porous network when the freeze–thaw process was used [34]. Montaser et al. used polyvinyl alcohol (PVA) with a concentration of 10% and sodium alginate (SA) with a concentration of 1% in a polymeric blend with n-isopropyl acrylamide for the treatment of reinfected wounds [35]. Rezaei et al. considered a polymeric blend of polyvinyl alcohol with a concentration of 12% and sodium alginate with a concentration of 4% utilizing nano-curcumin and graphene for wound healing [36]. Hong et al. used a polymeric blend of sodium alginate with a concentration of 2%, polyvinyl alcohol with a concentration of 3% involving chitosan (CS) with a concentration of 2% and prepared a polymeric blend for wound-healing applications [37]. Wei et al. reported that PVA and SA are compatible in polymeric blends and described that PVA and SA were miscible in any composition [38].

Curcumin shows anti-inflammatory, anti-mutation, anti-coagulant, anti-cancer, and anti-oxidative properties [39]. The source of curcumin is the plant Curcuma longa Linn, and curcumin is a natural polyphenol [40]. Zoi et al. [41] stated that curcumin has been found efficient in treating various types of cancer like lung, breast, gastric, pancreatic, prostate, brain, and leukemia. Kumari et al. [42] demonstrated that a nano-emulsion of curcumin led to accelerating burn wound healing by enhancing the pharmacological effects, acting as an anti-microbial agent. Hamilton et al. [43] suggested that the targeted delivery of curcumin achieved maximum bioavailability, even with lower amounts. After checking clinical trials, Shojaei et al. [44] concluded that nano-curcumin has positively impacted respiratory functions and inflammatory conditions in COVID-19 patients. Barchitta et al. mentioned that curcumin could play an effective role in wound healing, and curcumin can reduce the inflammation phase of wound healing [45]. Sood et al. reported that a blend of sodium alginate and curcumin has applications in cancer treatment and wound healing. This above-mentioned study demonstrates that sodium alginate has an advantage due to its ability to form a hydrogel, and curcumin was used due to its therapeutic effects [46].

In our research, a polymeric film made from the blend of polyvinyl alcohol and sodium alginate was prepared by following the solvent-casting technique. However, the main aim of this research was to design a complex blend made from more than two components for potential biomedical applications. For this purpose, curcumin and polydopamine were added to the polymeric blend of polyvinyl alcohol and sodium alginate. Polydopamine was used to modify the properties of the blend and protect the curcumin, which is light-sensitive. The composite with film-forming properties was studied for potential application in the wound-healing process. To the best of our knowledge, the composite proposed by us has not been studied before. Taking into account all the advantages of the single components, one can expect that the new composite may offer useful properties for biomedical applications.

## 2. Materials and Methods

Dopamine hydrochloride (PHR-1090, MW: 189.64), sodium alginate (W201502, CAS: 9005-38-3, MW: 396), and polyvinyl alcohol (363065, CAS:9002-89-2, MW: 146,000–186,000) were obtained from Sigma-Aldrich, Darmstadt, Germany. Curcumin (GP8291, CAS: 458-37-7, MW: 368.39) was obtained from Glentham Life Sciences, Corsham, United Kingdom. Ethanol (Cat. 32294, CAS: 64-17-5, MW: 46.07, 96%) was provided by Honeywell, Riedel-de Haen (Seelze, Germany). Aqueous ammonia (CAS: 1336-21-6, MW: 17.03, 25%) was obtained from POCH SA, Gliwice, Poland.

### 2.1. Preparation of Polydopamine

In this research, we prepared polydopamine using dopamine hydrochloride. Dopamine hydrochloride is toxic, so we needed to convert it to polydopamine. The polydopamine preparation procedure followed, as described by Gao et al. [47]. Dopamine hydrochloride (50 mg) was dissolved in 1 mL of distilled water. The dopamine hydrochloride dissolved in water was mixed with a combined solution of 9 mL of distilled water, 5 mL of ethanol, and 0.15 mL of aqueous ammonia in a flask. We put it to stirring at a speed of 100 rpm for 24 h. Stirring for 24 h was maintained to allow for the polymerization and oxidation of dopamine hydrochloride. As the dopamine hydrochloride, dissolved in the water, was combined with the ethanol, distilled water, and aqueous ammonia in the flask, a pale-yellow color was observed. With time, as the polymerization and oxidation proceeded, a color change was observed, and it started turning to a black color. After 24 h, the color observed was dark black, which confirmed the formation of polydopamine.

### 2.2. Preparation of Polymeric Blend of Polyvinyl Alcohol and Sodium Alginate

The polymeric blend of polyvinyl alcohol (PVA) and sodium alginate (SA) was prepared in a 50:50 proportion, and the polymeric film was prepared by following the solvent-casting technique. The PVA and SA solution concentration was 5% (*w*/*w*), and, from here, we name the blend PVA/SA.

### 2.3. Preparation of Polymeric Blend of Polyvinyl Alcohol, Sodium Alginate, and Curcumin

Curcumin is soluble in ethanol, and 5 mg of curcumin was dissolved in 5 mL of ethanol. We mixed the dissolved curcumin (Cur) with a concentration of 2% with a polymeric blend of polyvinyl alcohol (PVA) (concentration 5%) and sodium alginate (SA) (concentration 5%). This solution was kept under stirring for one hour. A polymeric film of PVA and SA containing curcumin (2%) was prepared by the solvent-casting method (PVA/SA/Cur).

### 2.4. Preparation of Polymeric Blend of Polyvinyl Alcohol, Sodium Alginate, Curcumin, and Polydopamine

Polydopamine was introduced in drops to the polymeric blend of PVA, SA, and curcumin (Cur). A polydopamine (PD) solution with a concentration of 2% was used. The solution was kept under stirring for one hour. Ultimately, polymeric films of PVA, SA, Cur, and PD were prepared following the solvent-casting method (PVA/SA/Cur/PD).

### 2.5. Microscopic Analysis

A microscopic analysis of the polymeric films was carried out with a Motic microscope (SMZ-171, China) at a 0.75 resolution. This optical microscope was used for the preliminary observation of the prepared polymeric films and the selection of the polymeric films to measure their mechanical properties.

### 2.6. Atomic Force Microscopy (AFM)

Atomic force microscopy was chosen to analyze the surface roughness of the polymeric films. An atomic force microscope (Veeso Digital Instrument, Santa Barbara, CA, USA) was used to perform the scans. The analysis was performed at standard room temperature and atmospheric pressure conditions. An area of 5 μm × 5 μm was selected for the scans. The computer program, NanoScope Analysis (Bruker, Ettlingen, Germany), was used to calculate the rough parameters.

### 2.7. Scanning Electron Microscopy (SEM)

Scanning electron microscopy (SEM) was utilized to determine the surface morphology of the polymeric films. A scanning electron microscope (LEO, Electron Microscopy Limited, Cambridge, UK) was used in this research.

### 2.8. Fourier Transform Infrared Spectroscopy

The Fourier-transform infrared (FTIR) spectra of the prepared polymeric films and curcumin powder were analyzed with a Nicolet iS10 spectrometer equipped with an ATR device (Thermo Fisher Scientific, Waltham, MA, USA). The range of 400–4000 cm^−1^ wavenumber was chosen to scan the samples at a 4.0 cm^−1^ resolution for 64 scans. The FTIR spectra were observed with the computer program Omnic 2009. An FTIR analysis was carried out to check the functional groups.

### 2.9. Mechanical Properties

The polymeric films were cut down into samples (5 cm × 1 cm), and their mechanical properties were tested with the Zwick & Roell 0.5 (Ulm, Germany) mechanical properties testing machine. The mechanical property results were recorded with the testXpert II 2017 computer program. The parameters used for the program were as follows: initial force maintained at 0.1 MPa, testing speed sustained at 50 mm/min, initial force speed upheld at 5 mm/min, and load cell kept with a value of 0.5 N. An ANOVA test was applied to analyze the mechanical property results statistically. This calculates the variance, standard deviation, and standard error.

## 3. Results

### 3.1. Microscopic Images

**Figure 1:**
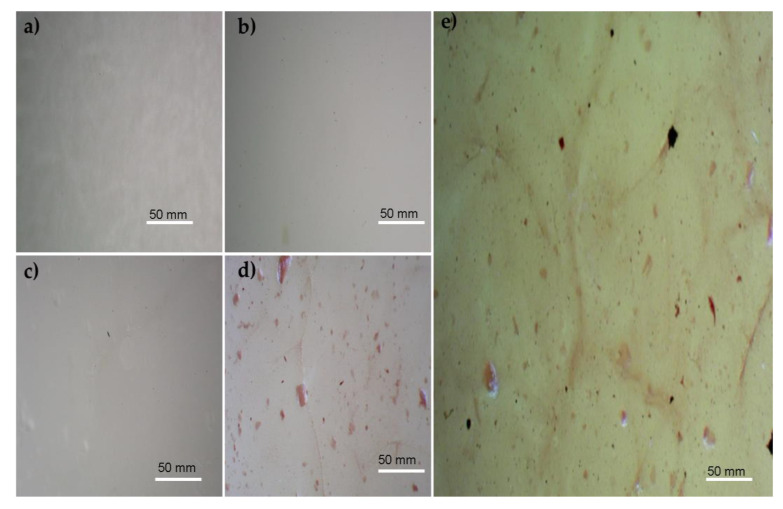
Microscopic images of films: (**a**) SA; (**b**) PVA; (**c**) PVA/SA; (**d**) PVA/SA/Cur; (**e**) PVA/SA/Cur/PD.

Microscopic images of the polymeric films are presented in Figure 1. The film obtained from the PVA solution with a concentration of 5% was observed, and the microscopic image is shown in Figure 1a. As can be seen, the PVA film shows a smooth and uniform morphology due to the PVA crystallinity [48].

The microscopic image of the films obtained from SA with a concentration of 5% is shown in Figure 1b. The microscopic image of SA presents a compact shape with irregularly shaped pores. Similar results regarding porosity were mentioned by Badita et al. [49]. The porous structure of the polymeric blend of PVA/SA has also been reported by other scientists, and one can find similar results in the existing literature [35,50,51]. As can be seen, the white pores and a few dots are similar to that in the SA film observed in Figure 1c.

Figure 1d represents the polymeric film containing PVA, SA, and Cur. The addition of curcumin led to the alteration of the film. The observed color change is due to the presence of curcumin, which presents a yellow color. As can be seen, the curcumin adhered to the porous network. Adhering curcumin to a SA/PVA porous network was compared with results in the scientific literature. Thanyacharoen et al. also reported a similar concept of attaching curcumin to the network of PVA/SA [52].

The polymeric film with PVA, SA, Cur, and PD was observed using a microscope. The microscopic image of PVA/SA/Cur/PD is presented in Figure 1e. It shows that the addition of polydopamine modified the polymeric film. After the PD addition, the black dots appear, which confirms the composite formation. The black dots can be noticed due to the polydopamine dark black color [53].

### 3.2. Atomic Force Microscopy (AFM)

**Figure 2:**
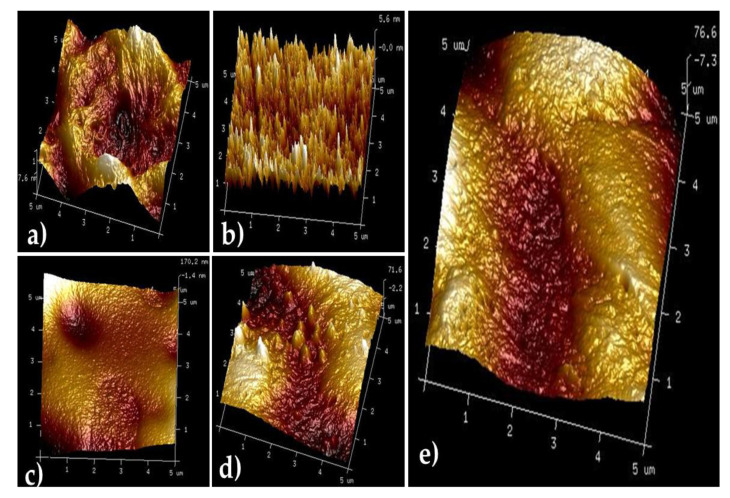
AFM images of (**a**)SA; (**b**)PVA; (**c**)PVA/SA; (**d**) PVA/SA/Cur; (**e**)PVA/SA/Cur/PD.

Atomic force microscopy (AFM) images of the polymeric films: PVA, SA, PVA/SA, PVA/SA/Cur, and PVA/SA/Cur/PD are presented in Figure 2.

The surface roughness in the form of the root mean square found with atomic force microscopy is presented in Table 1.

The polymeric film made of polyvinyl alcohol presented the lowest surface roughness, and surface roughness was found to be the highest among the studied specimens with SA. In the polymeric blend PVA/SA/Cur/PD, we observed smaller surface roughness than for SA but bigger than for PVA. This may indicate that components in the blend show miscibility that leads to the smoothness of the surface.

### 3.3. Scanning Electron Microscopy (SEM)

**Figure 3:**
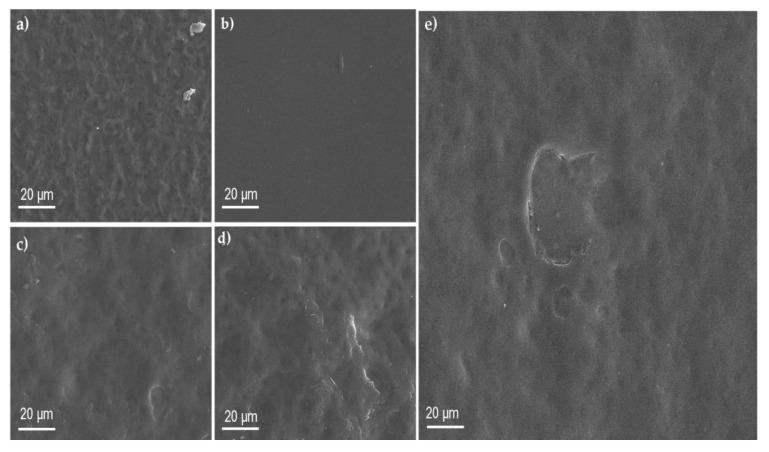
SEM images of films: (**a**) SA; (**b**) PVA; (**c**) PVA/SA; (**d**) PVA/SA/Cur; (**e**) PVA/SA/Cur/PD.

The SEM images of the polymeric films are shown in Figure 3.

A scanning electron microscopy (SEM) analysis of polyvinyl alcohol (PVA) presents a smooth, uniform, and non-porous structure. Shivakumara et al. also mentioned smooth, uniform, and non-porous structure results due to the crystallinity of the polyvinyl alcohol [48]. The SEM image of SA presents compact shapes, which show irregular pores, as mentioned by Badita et al. [49]. The polymeric blend PVA/SA image shows the porous network. Montaser et al. also analyzed the polymeric blend of PVA/SA with SEM, indicating the presence of pores [35]. Xie et al. prepared a polymeric blend of polyvinyl alcohol (PVA) and sodium alginate (SA) with a 5% concentration. They mentioned that SEM analysis shows the porous texture responsible for adhesion [50]. Mirzaie et al. also analyzed the SEM images of a polymeric blend of PVA/SA. They also reported the porous network of the polymeric blend [51]. Adding curcumin and polydopamine resulted in the modification of the surface properties of the PVA/SA blend. The SEM image of the PVA/SA/Cur and PVA/SA/Cur/PD are shown in Figure 3d,e. As can be seen, the surface smoothness was improved.

### 3.4. FTIR Spectrum

**Figure 4:**
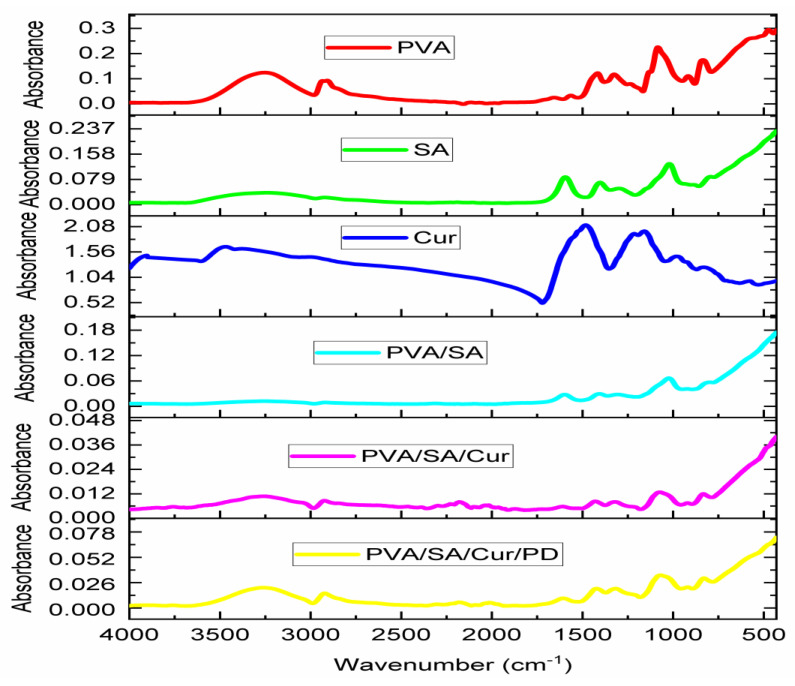
FTIR spectra of curcumin powder and polymeric blends.

The FTIR spectra of the polymeric blends with polydopamine and curcumin powder are presented in Figure 4.

The FTIR spectrum of the sodium alginate shows the characteristics band at 3242.cm^−1^, which is a result of the O-H stretching vibrations. The band at 1592 cm^−1^ is due to asymmetric -COO, and the band at 1405 cm^−1^ is the representation of the symmetric -COO resulting from the stretching vibration of the carboxyl groups [54]. The peak at 1025 cm^−1^ shows the C-O stretching vibrations. The peak at 949 cm^−1^ is an indication of the CO stretching, which indicates the presence of uronic acids. The peak at 882 cm^−1^ arises from the C1-H deformations, which are evidence of mannuronic acid residues. The peak at 814 cm^−1^ also indicates mannuronic acid residues [55].

The FTIR spectrum of the polyvinyl alcohol (PVA) indicates a band at 3262 cm^−1^, and the characteristic band was caused by the O-H stretching [56]. Awada et al. reported that O-H stretching results from intramolecular and intermolecular hydrogen bonding [57]. The peak at 2937 cm^−1^ resulted from the asymmetric stretching of CH_2_, and the peak at 2908 cm^−1^ resulted from the symmetric stretching of CH_2_. The peak with an intensity of 1660 cm^−1^ evolved from the water absorption [56]. The peak at 1567 cm^−1^ emerged from the stretching of C=C [58]. The bands with an intensity of 1416 cm^−1^ and 1325 cm^−1^ are attributed to the groups of CH and OH [59]. The peak at 915 cm^−1^ arises due to the CH_2_ rocking, and the peak at 833 cm^−1^ is caused by the C-C stretching [60].

In the FTIR spectrum of the polymeric blend PVA/SA, a band at 3243 cm^−1^ caused by O-H stretching and a band at 2907 cm^−1^ resulting from the C-H stretching vibration were found [61]. The peaks of sodium alginate were observed in the spectrum of polymeric blends at the intensities of 1596 cm^−1^ and 1409 cm^−1^, resulting from the asymmetric and symmetric vibrations of the carboxylate groups [62]. The peak with an intensity of 817 cm^−1^ also confirms sodium alginate with the confirmation of the mannuronic acid residues. The peak at 2907 cm^−1^ also indicates the presence of polyvinyl alcohol, ascribed to the asymmetric stretching of the CH_2_.

On the FTIR spectrum of curcumin, we can see the sharp peak with an intensity of 3510 cm^−1^, which shows the presence of the hydroxyl group [63]. The strong peak at 1628 cm^−1^ is due to the vibration of the C=C and C=O [64]. The peak at 1509 cm^−1^ is evidence of the C=O stretching, and the peak at 1277 cm^−1^ emerged due to the presence of the C-O stretching [65]. The peak at 1152 cm^−1^ shows the result of the C-O-C stretching [66]. The peak appears at 1026 cm^−1^, and Boscariol et al. reported that the peak is caused by the presence of the ether functional group (C-O-C) [67]. The peak at 961 cm^−1^ was identified by El-Nahhal et al. as a benzoate trans-CH vibration, which could cause this peak [68].

The FTIR spectrum of polymeric blend PVA/SA/Cur/PD presented major peaks at 3270 cm^−1^, 2907 cm^−1^, 1609 cm^−1^, 1417 cm^−1^, 1319 cm^−1^, 1082 cm^−1^, 916 cm^−1^, and 829 cm^−1^. The peak at 1609 cm^−1^ indicates the polydopamine presence due to the stretching of C=C in the ring or due to the amine group, as reported by Sun et al. [69]. The peak at 1319 cm^−1^ is evidence of curcumin, as mentioned by Ejaz et al. [70]. The peak at 1417 cm^−1^ shows the presence of sodium alginate due to the stretching of the carboxyl group in the form of the symmetric -COO vibration. The peak at 829 cm^−1^ is also evidence of sodium alginate because it shows the presence of mannuronic acid residues. The peak at 3270 cm^−1^ is due to the hydroxyl group, which shows the availability of the polyvinyl alcohol in the polymeric blend. The Fourier-transform infrared (FTIR) spectrum analysis confirmed the presence of polymeric compounds and the functional group’s availability.

### 3.5. Mechanical Properties

**Figure 5:**
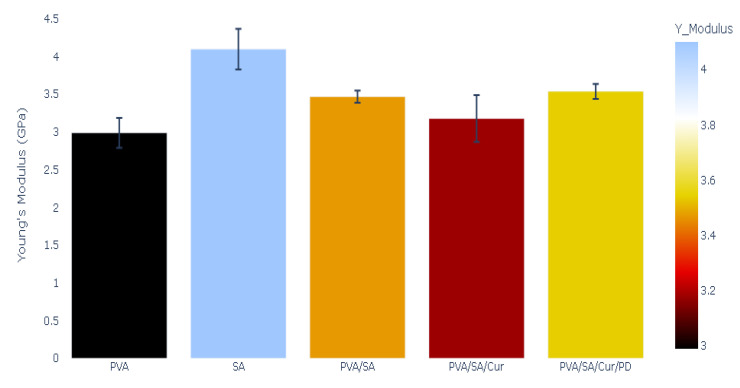
Young’s modulus of polymers and polymer blends and composites.

**Figure 6:**
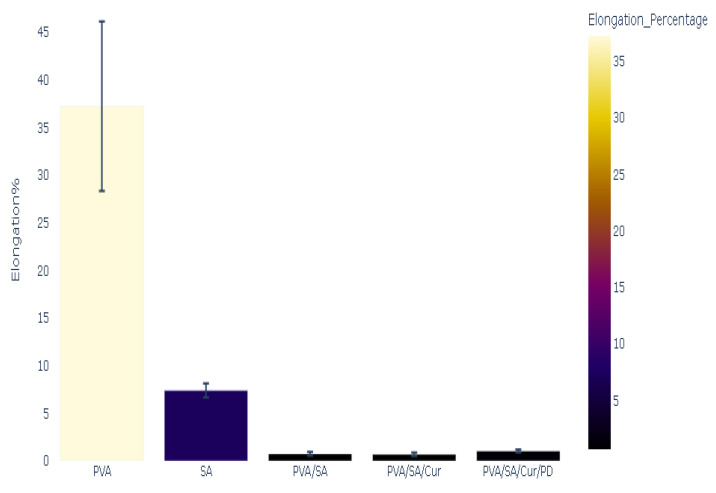
Elongation at break of polymers and polymer blends and composites.

**Figure 7:**
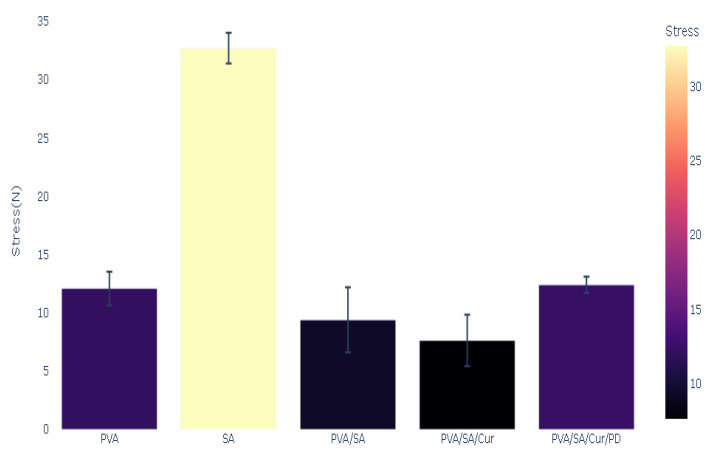
Stress of polymers and polymer blends and composites.

**Figure 8:**
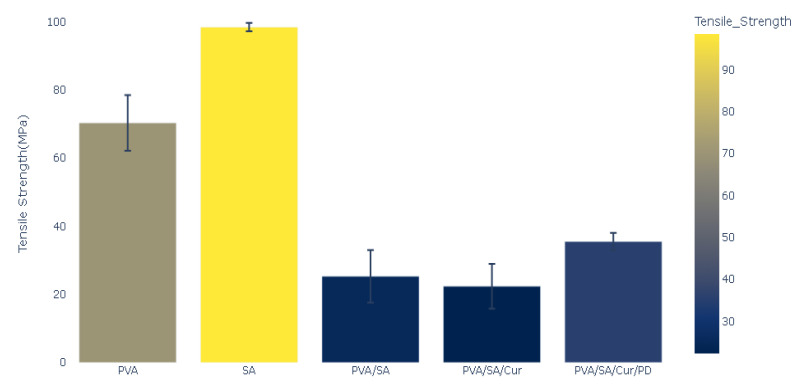
Tensile strength of polymers and polymer blends and composites.

The mechanical properties of the polymeric films were measured, and the results of Young’s modulus, stress, mechanical strength, and elongation percentage tests are presented in Figure 5, Figure 6, Figure 7 and Figure 8.

The value of Young’s modulus was higher in the case of the SA film than for the PVA film. It was 4.10 GPa for SA, whereas we recorded Young’s modulus of 2.99 GPa for PVA. Young’s modulus exhibited a value of 3.47 GPa for the polymeric blend of PVA/SA. The addition of curcumin to the polymeric blend of PVA/SA led to a further decrease in Young’s modulus to 3.18 GPa. Young’s modulus was 3.54 GPa for the PVA/SA/Cur/PD polymeric blend. Young’s modulus of the polymers is depicted in Figure 5.

The polymeric film of PVA presented the maximum elongation at break, with a value of 37.25%. The elongation at break was marked with a value of 7.4% in the case of the SA polymeric film. A decrease in the elongation percentage was observed for the polymeric blend PVA/SA and was only 0.75%. Adding Cur to a polymeric blend of PVA/SA decreased the elongation at break to 0.70% and showed minimal elongation at break. The addition of PD to a polymeric blend of PVA/SA/Cur resulted in a slight increase in the elongation at break to 1.05%, which can be checked in Figure 6.

The highest stress was noted for the SA film, with a value of 32.73 N, whereas the PVA/SA/Cur composite revealed the lowest stress value of 7.63 N. The stress value for the PVA was 12.09 N. Adding polydopamine to the basic mixture increased the stress value by 12.40 N. For the PVA/SA blend, the stress value was 9.40 N. The stress of the polymers is shown in Figure 7.

The comparative analysis of tensile strength for all the studied samples showed that the SA films had the highest tensile strength value of 98.48 MPa. The smallest value was found for the polymeric blend of PVA/SA/Cur, and in the case of the PVA film, the tensile strength value was 70.38 MPa. The PVA/SA/Cur composite manifested the lowest tensile strength value of 22.38 MPa. For the polymeric blend PVA/SA, the tensile strength value was 25.31 MPa, whereas, for the polymeric blend (PVA/SA/Cur/PD), it was 35.53 MPa. The values of the tensile strength of the polymers are displayed in Figure 8.

## 4. Discussion

Notably, using synthetic polymers in the biomedical field is much easier, but natural polymers have positive features due to their biocompatibility and biodegradability. Blending two or more polymers and/or biopolymers is another method of preparing polymeric materials for biomedical applications [71]. The blends of synthetic and natural polymers can form a new class of materials with improved mechanical properties and biocompatibility compared to single components. The possibility of polymer blending has, over the last three decades, caused increasing interest in new materials based on the blends of two or more polymers and composites. The new materials are predicted to be biocompatible while possessing good thermal and mechanical properties and applications in the biomedical field.

Our research used PVA, SA, curcumin, and polydopamine to fabricate polymeric blends and composites. Polyvinyl alcohol is a medically proven wound-healing material, and adding curcumin can enhance the anti-bacterial properties of a polymeric composite. Curcumin is also medically applicable due to its anti-bacterial effects. Polydopamine is a biopolymer obtained from dopamine. Dopamine is responsible in living organisms for feeling pleasure, satisfaction, and motivation. However, some derivatives of dopamine, e.g., dopamine hydrochloride, have toxic effects, so it needs to be converted to polydopamine to prevent negative dopamine effects in the polymeric composite. Polydopamine has non-toxic effects and can be used for biomedical applications, so, in this research, we added polydopamine to the polymeric composite of PVA, SA, and curcumin to fabricate polymeric films for potential wound-healing materials. Sodium alginate is utilized due to its amorphous nature, and it is preferred to be used in polymeric composites containing polyvinyl alcohol [28,33,34]. By mixing PVA, SA, Cur, and PD, we can obtain stable polymeric films with mechanical properties sufficient for biomedical applications. The slight deterioration in mechanical properties in the polymer blends after curcumin addition may be the result of a disruption in hydrogen bonding between components. Slight shifts of bands in the FTIR spectra suggest that the functional groups of polymers may be engaged in hydrogen bonding, and after mixing, new hydrogen bonds between different polymers can be formed. Interactions between components in our composite may lead to alterations in the surface structure and the bulk. To fabricate an appropriate polymer film for medical applications by the following solvent-casting method, the concentration should be properly chosen. The concentration of the polymers used for the fabrication of polymeric blends in our research was decided based on the film-forming properties of a single polymer and previous research done by us and reported by other researchers [33,34,35,36,37,38]. The morphology of the composite depends on interactions between components in the blend. In our case, the addition of curcumin to the PVA/SA blend led to the deterioration of mechanical properties. However, by adding polydopamine we could improve the mechanical characteristics of the PVA/SA/Cur composite, and we have achieved sufficient mechanical properties. Newly developed polymeric materials based on the blends of PVA, SA, Cur, and PD should be biocompatible because the single components used in this research are biocompatible. We have not used toxic solvents nor cross-linking agents to prepare the blends, so it is expected that the materials can be applied in the biomedical field. However, to make the composites effective, new biomedical materials for wound healing—not only biocompatibility—should be proved as well as the possibility of material sterilization.

Although there is much research regarding polymeric blends for wound-healing applications, few polymer blend-based hybrid dressings are under clinical trials. According to the literature, hybrid polymeric blends can form promising wound dressings. Moreover, several active compounds can be incorporated into polymeric matrices. Different strategies may enhance their bactericidal activity and wound-healing effects, such as incorporating bioactive agents like nanoparticles, plant extracts, antibiotics, growth factors, etc.) into the prepared wound dressings. As curcumin and polydopamine show anti-bacterial properties, we can expect that the composite materials we proposed have great potential for further in vitro and in vivo studies to be applied in wound-healing treatment. Moreover, polydopamine has the ability of biological functionalization or bioconjugation, so further modifications are possible.

## 5. Conclusions and Future Outlook

Polymeric films based on polyvinyl alcohol and sodium alginate have been successfully developed. FTIR spectra confirmed the structure of the blend. The film’s surface showed a porous structure, but the addition of curcumin caused an improvement in the surface properties. Adding curcumin did not improve the mechanical properties but, when polydopamine was added, the mechanical properties were good enough for application as wound-healing materials. The surface roughness was much higher in the polymeric film of sodium alginate than in the polyvinyl alcohol. The addition of curcumin to the polymeric blend of PVA/SA led to a reduction in the surface roughness. Adding polydopamine to the polymeric blend led to a further decrease in surface roughness and showed an improvement in surface smoothness. The surface roughness plays a pivotal role in the preparation of wound-healing materials, so, depending on the kind of wound, the amount of addition can further modify the surface properties. Although we can expect biocompatibility of newly developed polymeric materials, this should be proved in the future. Moreover, in the future, prepared composites can be linked to natural compounds to enhance their biological properties.

## Figures and Tables

**Table 1:** Surface roughness of polymeric films analyzed by AFM.

Polymer Films and Composites	Rq (nm)	Ra (nm)
SA	66.6	53.4
PVA	1.6	1.26
PVA/SA	37.4	22.5
PVA/SA/Cur	25.7	21.9
PVA/SA/Cur/PD	25.1	21.1

## Data Availability

The data is reported within the article.
